# Unmet Needs in Dystonia: Genetics and Molecular Biology—How Many Dystonias?

**DOI:** 10.3389/fneur.2016.00241

**Published:** 2017-01-16

**Authors:** Dineke S. Verbeek, Thomas Gasser

**Affiliations:** ^1^Department of Genetics, University Medical Center Groningen, University of Groningen, Groningen, Netherlands; ^2^Department of Neurodegenerative Diseases, Hertie Institute for Clinical Brain Research, University of Tübingen, and German Center for Neurodegenerative Diseases (DZNE), Tübingen, Germany

**Keywords:** dystonia, genetics, GWAS, whole-exome sequencing, variant validation

## Abstract

Genetic findings of the past years have provided ample evidence for a substantial etiologic heterogeneity of dystonic syndromes. While an increasing number of genes are being identified for Mendelian forms of isolated and combined dystonias using classical genetic mapping and whole-exome sequencing techniques, their precise role in the molecular pathogenesis is still largely unknown. Also, the role of genetic risk factors in the etiology of sporadic dystonias is still enigmatic. Only the systematic ascertainment and precise clinical characterization of very large cohorts with dystonia, combined with systematic genetic studies, will be able to unravel the complex network of factors that determine disease risk and phenotypic expression.

## Introduction

Compared to many other neurologic diseases, the dystonias have only relatively recently been recognized as a group of somatic disorders with a characteristic spectrum of clinical manifestations and pathophysiological features. Although disturbed signaling of basal ganglia circuits have soon been identified as the major neurophysiologic substrate, in the absence of visible pathology on autopsy, the cause of what then was called “primary” dystonia, be it focal or generalized, with or without additional neurologic abnormalities, remained completely enigmatic. It was only the discovery of the torsinA (*TOR1A*) gene in 1997 (1) as the major cause for primary generalized dystonia, traditionally also called “Oppenheim’s dystonia,” that promised to shed more light on the molecular events leading to dystonic syndromes, and thus, the ultimate cause of at least a subgroup of dystonic disorders. Yet, despite the considerable progress that has been made in the years since this seminal discovery in the dissection of the genetic basis of dystonia, in the elucidation of molecular pathways and the development of animal models, it must be admitted that the path from mutation to disease is still poorly, if at all, understood, and thus, interventions based on a deeper understanding of the molecular pathophysiology of the dystonias are still lacking.

In the absence of this understanding, genotype–phenotype correlations were expected to help to understand the relationship between the molecular and clinical sphere by describing a more or less unequivocal clinical presentation to be associated with, and causally related to a specific genetic variant or mutation. While this has been relatively easy for DYT1-dystonia, with TOR1A mutations causing a rather specific phenotype of early-onset primary generalized dystonia with limb involvement, or in DYT11, the form of myoclonus-dystonia (M-D) caused by mutations in the *SGCE* gene (2), it has become clear that in most other cases, genotype–phenotype correlations are much more variable and complex than initially believed and that genetic classifications do not translate one to one to clinical phenotypes. Mutations in some dystonia genes, such as *THAP1* (DYT6), can cause both focal/segmental and generalized forms of the disease, while others (e.g., TH or GCH1) may even give rise to both isolated and combined dystonias (3). The new classification of dystonias proposed by Albanese et al. takes these issues into account by clearly separating two “axes” of classification, i.e., clinical phenotype and etiology (4).

In addition, the vast majority of patients with the more common forms of dystonia, such as cervical dystonia, blepharospasm, or writer’s cramp, have no or only an uninformative family history without clear Mendelian inheritance pattern. Moreover, issues including non-penetrance and variable expression of mutations even further complicates the distinction between complex and heritable forms of dystonia and will be discussed at the end of this chapter. Thus, the role of genetic factors in the etiology of these forms is still unclear, which has great impact on genetic counseling of patients and relatives. Nevertheless, the recognition of a small group of patients in whom a dystonia syndrome clearly is inherited as a monogenic trait (Table 1) allowed first “genetic entry points” and is beginning to give insight into the molecular pathogenesis of the disorder.

**Table 1:** **List of genes for monogenic forms of isolated and combined dystonias**.

Locus	Disease	Type	Inh.	Gene name	Chrom.
DYT1	Oppenheim’s torsion dystonia	ID	AD	*TorsinA*	9q34
DYT2	Early-onset recessive TD	ID	AR	*HPCL*	1p35
DYT3	Lubag (x-linked dystonia-parkinsonism)	CD	X-R	*TAF1*	Xq13.1
DYT4	Whispering dystonia (one family only)	ID	AD	*TUBB4*	–
DYT5a/b	Dopa-responsive dystonia	CD	AD	*GCH1, TH, SPR*	14q22.1
DYT6	Craniocervical dystonia (Mennonite/Amish)	ID	AD	*THAP1*	8q21-q22
DYT7	Familial torticollis	ID	AD	–	18p
DYT8	Paroxysmal non-kinesigenic choreoathetosis	ID/CD	AD	*MR1*	2q33-q35
DYT9	Paroxysmal dyskinesias with spasticity	CD	AD	*GLUT1* (*SLC2A1*)	1p21
DYT10	Paroxysmal kinesigenic dyskinesia	ID/CD	AD	*PRRT2*	16p11.2
DYT11	Myoclonus-dystonia	CD	AD	*e-SG*	7q21.3
DYT12	Rapid-onset dystonia-Parkinsonism	CD	AD	*ATP1A3*	19q13
DYT13	Craniocervico brachial	ID	AD	–	1p36
DYT15	Myoclonus-dystonia	CD	AD	–	18p11
DYT16	Dystonia-Parkinsonism	CD	AR	*PRKRA*	2q31.2
DYT17	Juvenile-onset TD with torticollis and dysarthria	CD	AR	–	20p11
DYT18	Paroxysmal exercise-induced dystonia	ID/CD	AD	*GLUT1* (*SLC2A1*)	1p31
DYT19	Paroxysmal kinesigenic dystonia 2	ID/CD	AD	–	16q13
DYT20	Paroxysmal non-kinesigenic dystonia 2	ID/CD	AD	–	2q31
DYT21	Pure dystonia, mixed distribution	ID	AD	–	2q14
DYT23	Cervical dystonia/myoclonus-dystonia	ID	AD	*CACNA1B*	9q34
DYT24	Mixed dystonia	ID	AD	*ANO3*	11p14
DYT25	Cervical dystonia	ID	AD	*GNAL*	18p11
DYT26	Myoclonic dystonia	CD	AD	*KCTD17*	22q12
DYT27	Cervical/limb/generalized	ID	AR	*COL6A3*	2q37
	Cervical dystonia	ID	AD	*CIZ1*	9q34

*Inh., inheritance mode; Chrom., chromosomal region*.

*The assignment of a DYT number does not mean that the pathogenic role of mutations in the listed genes is unequivocally confirmed*.

## Novel Genes and Genetic Risk Factors for Dystonias

The genetic analysis of the dystonias presents a number of challenges. At first glance, the presence of a genetic mutation seems to be an undisputable and objective finding in a patient with a familial neurologic disease. On closer inspection, however, the causative role of DNA-sequence variants of a given gene is often not easy to elucidate. This is why today the more neutral term “genetic variant” is often preferred to the term “mutation,” which carries the connotation of being “harmful.” Today it is clear that by far not all genetic variants, even in a gene known to be linked to a specific inherited disease, are in fact disease causing. Many cases with familial dystonia and the vast majority of sporadic cases cannot be explained by one or multiple presently recognized and validated mutations in known dystonia genes. Two technologies are promising to rapidly fill this knowledge gap: (i) whole-exome sequencing (WES) and (ii) genome-wide association studies (GWAS). While GWAS are designed to detect common (>5% in the population) genetic variants that usually reside in non-coding (presumably regulatory) regions of the genome and exert only a relatively small effect on disease risk, WES targets (usually rare) coding variants that are more likely to have a damaging effect on protein function.

### WES in Dystonia

The part of the human genome that codes for proteins, the coding sequence (also called the “exome,” as it is the sum of all exons), spans about 50 million base pairs. It contains several million rare to very rare variants from the consensus sequence, about 20,000 in any given individual. More than half of them lead to an alteration in the amino acid sequence of the encoded proteins (5), and thus can potentially change protein function. A comprehensive cataloging of these variants and the elucidation of their functional consequences is a daunting task. Their low frequency (usually <1% in the population, often much lower) requires the study of very large patient cohorts, and the fact that their effect strength is likely to be only moderate (in other words, their penetrance on any phenotypic readout is incomplete) means that they can be also detected, although at even lower frequencies, in asymptomatic individuals. Also, the occurrence and frequency of those rare variants varies considerably between populations. This means that rare-variant association testing have to be done using carefully matched patient/control cohorts. Finally, it is very likely that interaction within functional gene networks plays an important role in determining overall function, a level of complexity that has not yet been addressed in most cases. Sophisticated bioinformatics analyses taking into account all these aspects will be necessary to make sense of the enormous amounts of data generated by WES.

The question of how to validate a potentially pathogenic mutation is of course not restricted to the dystonias or to inherited neurologic diseases. Guidelines have been published to establish a standardized workflow to assess the causal role of detected variants (6), but the issue remains challenging and many variants will have to be classified as “of unknown significance.” Four lines of evidence can support a pathogenic role of a detected variant of which each has its merits and its limitations.

(1) Genetic evidence: co-segregation in a large family with demonstration of the variant in multiple affected and its absence in unaffected family members remains the most stringent proof of pathogenicity. Unfortunately, sufficiently large families are rare, and reduced penetrance or non-genetic phenocopies may hamper these analyses. Alternatively, a statistically significant enrichment of the variant in question in multiple patient cohorts as compared to unaffected controls may serve as genetic evidence for pathogenicity. Very large cohorts may be necessary to reliably assess the role of rare or very rare variants (with population frequencies of ≪0.1%).
(2) Population evidence: the frequency of an increasing number of genetic variants in the population is publicly available in a number of databases, such as the exome variant server (EVS^1^) or the ExAC database.^2^ It is generally assumed that variants that are found at a relatively high frequency in such databases (e.g., >0.1%) are unlikely to be disease causing with high penetrance in rare diseases, because this would be incompatible with the epidemiology of these disorders. The limitation of these databases however is that they are derived from a collection of genetic exome sequencing studies, i.e., from patient cohorts with different diseases and from different countries. This clinical and demographic information is usually not available to users of the database, thus introducing unknown biases.
(3) *In silico* analysis: freely accessible computer programs have been developed to assess the functional consequences of DNA variation, such as PolyPhen^3^ or SIFT^4^ or mutation taster.^5^ They are usually based on the analysis of phylogenetic conservation (assuming that a change of a highly conserved amino acid is more likely to be deleterious than more evolutionarily variable ones) or on the predicted biophysical consequences of an amino acid exchange. While those programs are of value, they are best used to provide guidance for further studies, rather than to use them to assign a pathogenetic role to a variant in the setting of clinical testing, because their reliability is still in question.
(4) Functional analyses: functional analyses in cellular or animal models can provide insight into the consequences of a coding mutation of a gene and can even allow to identify drug targets and promising lead compounds for correcting the dysfunction caused by a mutation. A good example is the electrophysiological analysis of mutations in ion channel genes using patch clamp techniques in the Xenopus oocyte system. However, in the majority of cases, the relevant cellular function of a gene product is unknown, and thus, it is also unclear if the readout in an artificial model system is relevant to the disease under question. The generation of transgenic mouse models used to be a time-consuming and costly procedure; however, the availability of the clustered regularly interspaced short palindromic repeats (CRISPR)-associated RNA-guided endonuclease Cas9 (CRISPR-Cas9) technology opens up new avenues for genetic follow-up work as it allows for relatively rapid and efficient screening for loss of functional consequences (7). Furthermore, in addition to the generation of loss of function alleles, this technology can be used to introduce human mutations directly in the genome of mice creating mouse models mimicking human disease and multiple genes can be edited at the same time allowing studying gene–gene interactions.

So far, WES has already successfully facilitated the discovery of several new rare Mendelian dystonia genes (8). For example, the genes *GNAL* and *ANO3* have been identified using exome sequencing approaches in large families showing clear segregation of the mutation with the disease (9, 10). Potentially pathogenic variants in these genes have also been found in other, independent cohorts (11, 12), which are absent from public exome databases, and electrophysiological studies suggested plausible functional changes in ion channel and second messenger function, thus fulfilling all the criteria for pathogenicity, as described above. However, the recent findings highlight the challenge for future research with the notion that several of the recently reported genes, e.g., *CIZ1* and *COL6A*, found in families with autosomal dominant and recessive isolated dystonia with cervical predominance, respectively (13, 14), could not be unequivocally confirmed by other groups (15).

### GWAS in Dystonia

While exome sequencing aims to identify rare variants thought to be disease causing in familial isolated or combined dystonias with moderate to high penetrance, GWAS are beginning to explore the role of common genetic variability as risk factors for sporadic dystonia. Most common variants are located not in the coding region of the genome, the exome, but rather in non-coding intronic or intergenic regions, or in 3′- or 5′-untranslated regions of genes. They do not alter the protein sequence, but rather are thought, if they are functional, to modify the expression, the regulation, or the use of alternative splice variants of nearby (sometimes also far away) genes. Notably, this gene selection may be biased for regions that contain multiple genes and have high linkage disequilibrium.

GWAS have been extremely successful in identifying dozens of risk loci for many neurologic diseases, such as Parkinson’s disease, Alzheimer’s disease, or multiple sclerosis (16–18). Usually, very large cohorts, in the range of thousands or tens of thousands of patients and controls, are needed to reliably detect these risk loci, because their individual effects are usually very small, increasing the risk to develop the disease under investigation by a factor of 1.1–1.5. Thus, this information cannot be used for individual genetic counseling, but rather is expected to provide insight into the molecular networks underlying the pathogenesis of complex disorders. So far, only relatively small GWAS have been undertaken in dystonic syndromes. Lohmann et al. decided on focusing on a very specific phenotype, musician’s dystonia (MD), a form of the disease that affects 1–2% of professional musicians, speculating that this restriction would lead to a greater homogeneity of the patient sample and thus facilitate the detection of risk variants. They found common variants in the arylsulfatase G (*ARSG*) gene in a two-stage design, interrogating cohorts of 127 MD patients and 984 controls in the exploratory and 116 patients and 125 healthy musicians in the confirmation cohort (19). A single intronic variant was identified in an intron of *ARSG* (rs11655081; *P* = 3.95 × 10^−9^); odds ratio, 4.33; 95% confidence interval, 2.66–7.05. This variant was also associated with sporadic writer’s cramp, a subtype of dystonia thought to be closely related to musician’s dystonia (*P* = 2.78 × 10^−2^), but not with any other focal or segmental dystonia. ARSG hydrolyzes sulfate esters and is among others involved in protein degradation (20). The fact that dogs carrying homozygous mutations or dogs deficient for ARSG develop ceroid lipofuscinosis and accumulate heparin sulfate in visceral organs and central nervous system leading to behavioral deficits (21, 22) made ARSG an attractive disease gene for musician’s dystonia. In an attempt to identify the causal mutation in *ARSG*, the coding region of *ARSG* was screened for mutations using Sanger sequencing in Dutch Writer’s Cramp and German Musician’s Cramp cohorts (23). Variant rs61999318 (p.Ile493Tyr) was significantly enriched in Writer’s Cramp cases compared to European Americans in the EVS database (*P* = 0.0013), but no conclusive mutation was identified. Additionally, an overall enrichment for rare, protein-changing variants was observed in Writer’s Cramp cases compared to controls (*P* < 0.01), validating a role of *ARSG* in Writer’s Cramp.

In another GWAS, Mok et al. compared 212 cases with cervical dystonia with 5,173 controls (24). No single SNP was found to be associated on a genome-wide significance level (5 × 10^−8^), but one variant was found to be suggestive near exon 1 of a gene for a sodium leak channel (*NALCN*) with a *P*-value of 9.76 × 10^−7^. Dysfunction of such an ion channel is a plausible risk factor for dystonia, but in another small study, Gómez-Garre et al. were unable to confirm this association (25). The lack of association may be due to the fact that not all regions of the genome are equally covered, or that despite the large sample size there is lack of power to detect low effects, as is often the case for most associated or the associated gene identified by GWAS. In the end, only very large GWA studies including thousands of samples or large meta-analysis will be able to unequivocally resolve this issue.

## Example for Primary Generalized Dystonia (DYT1)

Primary generalized dystonia (Oppenheim’s dystonia) is the prototype of a Mendelian form of the disease. It is most frequently, and possibly caused by a single specific mutation, a deletion of a single GAG triplet encoding a glutamate residue, in exon 5 of a gene, *TOR1A* (1), encoding an ATP-binding protein called TorsinA. The protein appears to have chaperone function and is located in the nuclear membrane and the endoplasmic reticulum, but its precise function is still unknown, and it is completely unclear why the loss of a glutamate residue, which is the result of the disease-causing GAG deletion mutation, leads to dystonia. Even in a relatively “simple” case such as DYT1-dystonia, there remain many unanswered questions with respect to genotype–phenotype correlations, for example, (i) what determines penetrance of the DYT1-mutation, i.e., why do some mutation carriers develop the clinically manifest disorder, while many others do not and (ii) can mutations other than the classic GAG deletion cause dystonia? The answer to both questions could provide important insight into the molecular events leading from mutation to disease.

### Modifiers of the DYT1 Phenotype

Clinically, DYT1-dystonia presents almost always before the age of 20 years with an onset in a leg or arm, and progresses, not always, but in most cases, to a severe generalized form of the disorder (26). Remarkably, only about 30% of carriers of the disease-causing GAG deletion develop the disorder, suggesting the presence of genetic or non-genetic modifiers. Once the critical age is passed, a disease manifestation becomes unlikely, in contrast to neurodegenerative disorders. While reduced penetrance is the rule, rather than the exception in all neurogenetic disorders including DYT1-dystonia, its determinants are largely unknown, but can at least, in part, be explained by genetic modifiers. In addition, genetic modifiers may also account for differences in phenotypic expression of the GAG deletion leading to less common DYT1-dystonia phenotypes including late age of onset, focal, or segmental phenotypes and involvement of the craniocervical muscles (27).

**Figure 1:**
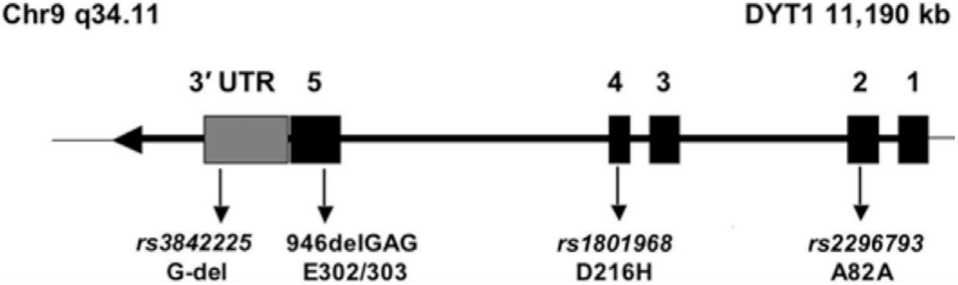
**Genomic structure of the *TOR1A* gene**. The penetrance of the 946del GAG mutation in exon 5 (E302/303) is modified by polymorphism, rs1801968 (p.Asp216His). Reprinted from Ref. (28).

One of such a genetic modifier variant involved in reduced penetrance is a relatively common coding polymorphism in the *DYT1* gene that affects nucleotide 646 of the cDNA sequence. The respective codon encodes the amino acid aspartate (D) at position 216 of the protein (p.216D) (see Figure 1). About 85% of all chromosomes in a normal population carry the wild-type nucleotide guanosine, while approximately 15% of chromosomes carry the variant cytosine at this position, encoding the amino acid histidine (H). In itself, this polymorphism has no known functional consequence.

However, carriers of the rare histidine variant *in trans*, i.e., on the allele, which does not carry the disease-causing GAG deletion, appear to be protected from the deleterious consequences of the disease-causing mutation of the *TOR1A* gene. Risch et al. studied a cohort of manifesting and non-manifesting carriers of the GAG deletion and found that only about 3% of those with the H-allele will eventually show symptoms of dystonia, while penetrance is slightly increased, from 30% in all GAG deletion carriers, to 35% in those carrying the more common aspartate at position 216 (28). Obviously, this can explain only a small fraction of the total phenotypic variability, because the protective variant is relatively rare in the population, but it may serve as an example for other diseases and modifiers. A better understanding of this functional interaction could also provide interesting hints toward possible therapeutic targets.

### Do Non-GAG Deletion Mutations in TOR1A Cause Dystonia?

The more widespread use of gene sequencing in dystonia patients has more recently uncovered a number of additional variants in *TOR1A* in patients with isolated dystonia. This poses the question if there is more than one genetic form of DYT1-dystonia. In a recent review, Dobričić et al. identified eight non-GAG deletion mutations in dystonia patients, most of them occurring in sporadic patients with adult onset forms of dystonia (29). This paper raises the important problem, how to validate the pathogenicity of rare variants detected by targeted or WES.

For *TOR1A*, for example, more than 100 rare variants are documented in the ExAC database, among them the p.Val129Ile change that was found in a patient with adult onset cervical dystonia by Dobričić et al. (29). While the prediction programs assigned the variant the status of “probably disease causing,” and it was very rare in the ExAC database (9/121.412 alleles), genetic evidence from the family was not supportive (negative family history, three unaffected mutation carriers). Population data from matched cohorts with and without dystonia or functional assays are not available. Thus, the pathogenic role of this variant will remain uncertain, and the same is true for other variants in *TOR1A*.

## Examples of Combined Dystonia (Previously “Dystonia-Plus” Syndromes)

Dystonia may also co-occur with additional movement disorders such as Parkinsonism or myoclonus and are then referred to as M-D or dystonia with Parkinsonism (e.g., in dopamine-responsive dystonia). In exceptional cases, the dystonia is accompanied by other neurological or systemic disorders but these are beyond the scope of this article.

### Mutations in *SGCE* Cause Myoclonus-Dystonia

Myoclonus-dystonia (M-D) is characterized by the combination of focal or segmental dystonia presented as cervical dystonia and/or writer’s cramp and shock-like jerks most often affecting neck and upper limbs, whereas the legs are less affected. The myoclonic jerks can be significantly reduced by the consumption of alcohol. The onset of disease occurs most often in childhood but the symptoms can also present in early adulthood. The suggested prevalence of MD is about two per million in Europe (30). The disease is inherited in an autosomal dominant fashion and up to ~30% of the MD cases carry loss of function mutations in *SGCE* encoding epislon sarcoglycan (DYT11) (2), indicating that not all *SGCE* mutations are identified using the current technologies and that additional MD genes can be found. The identification and functional characterization of these novel genes will increase our understanding of the underlying molecular biology of MD.

Genetic diagnostics of MD is sometimes complicated by the fact that *SCGE* undergoes maternal genomic imprinting, and markedly reduced penetrance is observed in affected families in which the mutant allele is silenced when inherited from the mother (31). Additionally, extended and complex MD phenotypes including cavernous cerebral malformations, hearing loss, and dysmorphisms may be the result of the fact that *SGCE* is sometimes deleted together with neighboring genes including *COL1A2* encoding the collagen alpha-2(1) chain reflecting haploinsufficiency of both *SGCE* and *COL1A2* (32). Patients with dominant negative mutations in *COL1A2* are linked to osteogenesis imperfecta types I–IV, Ehlers-Danlos syndrome type VIIB, recessive Ehlers-Danlos syndrome classical type, idiopathic osteoporosis, and atypical Marfan syndrome (33), whereas haploinsufficiency of *COL1A2* leads to milder phenotypes.

### Novel Myoclonus-Dystonia Genes

In 2002, a second MD locus was mapped to chromosomal region 18p11 (DYT15) in a large Canadian family of whom all cases were affected by myoclonus and four also displayed limb-dystonia (34). Yet, up to date, this finding was not replicated nor was the disease gene identified. This further demonstrates the genetic heterogeneity of the dystonias. Additionally, given the notion that exome sequencing in linkage intervals is quite successful suggests that either the mutation is missed due to (1) low coverage of DNA reads as, for example, the mutation may be present in a repeat-dense region, (2) the mutation is not covered by the exome capturing kit, or (3) the mutation is non-coding.

The successful combination of linkage analysis and exome sequencing was recently demonstrated by the identification of the disease genes for DYT23 and DYT26 (35, 36). In DYT23, a p.Arg1389His variant in *CACNA1B* encoding the voltage-gated calcium channel Cav2.2 was reported to cause MD plus in a large Dutch family. *Cacna1b* null mice exhibit a hyperkinetic movement disorder (37) and mutations in the homologous region of *CACNA1A* (Cav2.1), the subunit that together with Cav2.2 controls depolarization-induced calcium entry and transmitter release, were already reported to cause episodic ataxia and/or familial hemiplegic migraine (38). These findings support a role of Cav2.2 in the etiology of MD. Equally important, the p.Arg1389His variant fulfilled the majority of the four lines of evidence to support a pathogenic role. The variant (1) segregated in the family with the disease, (2) the MAF of the variant is <0.1% [carrier frequency of 0.0003809% in the EXAC browser (assessed January 2016)], (3) the variant was *in silico* predicted to be damaging, and (4) the variant affected channel functioning as was demonstrated by extensive electrophysiological studies in cell models. However, no second family carrying mutations in *CACNA1B* was yet identified and screening of an additional large European multicenter cohort of MD cases for the presence of the p.Arg1389His variant yielded identical frequencies in cases versus controls (39). Additionally, given that *CACNA1B* seems like the perfect candidate gene for this dystonia-plus syndrome, three other variants segregated with the disease in this family, the pathogenicity of the p.Arg1389His variant remains to be further confirmed *in vivo*.

In DYT26, two independent MD families of different ethnic background carried a recurrent mutation in *KCTD17* (p.Arg145His) encoding the potassium channel tetramerization domain containing 17 (36). This variant was not reported in ExAC, but variant p.Arg145Cys was observed with an allele frequency of 0.00001656. *KCTD17* was shown to operate in a large gene cluster involved in calcium-homeostasis and aberrant endoplasmatic reticulum calcium signaling was observed in fibroblast of patients. Both the genetic findings for DYT23 and DYT26 suggested alterations in calcium as pathomechanism underlying MD.

## Examples of Dystonia Combined with Parkinsonism

### Dopa-Responsive Dystonia

Dopa-responsive dystonia is characterized by a childhood onset dystonia with diurnal fluctuations (36). Importantly, the dystonia can be ameliorated by l-dopa treatment and Parkinsonism can occur later in the disease stage. Dopa-responsive dystonia also exhibits reduced penetrance, with unknown origin. Notably, the first disease gene that was identified to underlie a Mendelian form of dystonia was GTP cyclohydrolase (*GCHI*) (36). Mutations in *GCHI* led to autosomal dominant inherited dopa-responsive dystonia (DYT5a) and *GCHI* mutation carriers present with a childhood dystonia, but adult disease can mimic Parkinson’s disease. Up to date, more than 100 different mutations have been identified throughout the coding region and 5′UTR region of *GCHI* and no clear genotype–phenotype correlation could be established. However, some of the mutations may predispose to the risk to develop Parkinson’s disease (40). By sequencing, mutations in *GCHI* are found in only 40–60% of the dopa-responsive dystonias, leaving a large fraction of the patients genetically undiagnosed. GTP cyclohydrolase is involved in the production of an essential cofactor for biosynthesis of monoamine neurotransmitters, and additionally mutations in other enzymes leading to deficiency in the dopamine synthesis were reported to cause dopa-responsive dystonia, including tyrosine hydroxylase (*TH*), sepiapterin reductase (*SPR*), and 6-pyruvoyl tetrahydrobiopterin (*PTP*) synthase (41). Patients with mutations in these genes present with more severe and complex clinical pictures compared to heterozygous *GCHI* mutation carriers. Notably, cases with mutations in genes outside the dopamine synthesis pathway such as *ATXN3* causing spinocerebellar ataxia type 3 or SPG11 underlying spastic paraplegia type 11 can manifest as a dopa-responsive dystonia (42, 43), thereby broadening the clinical and genetic spectrum. With the introduction of exome sequencing in the clinic, novel disease genes underlying dopa-responsive dystonia will be identified.

### Rapid-Onset Dystonia-Parkinsonism

Rapid-onset dystonia-Parkinsonism is characterized by a sudden onset of dystonia often accompanied with Parkinsonism within hours or weeks induced after mental stress or physical trauma. The disease is inherited in an autosomal dominant manner with reduced penetrance. Heterozygous missense and *de novo* mutations in the Na+/K+-ATPase alpha3 subunit (*ATP1A3*) can cause either rapid-onset dystonia-parkinsonism (DYT12) (44), or alternating hemiplegia of childhood (AHC), a severe neurodevelopmental syndrome characterized by hemiplegic episodes and neurological complaints (45), respectively. Notably, no *DYT12* mutations were reported to cause AHC, whereas in two cases the same amino acid was affected. In contrast, some AHC cases were reported to develop late-onset rapid-onset dystonia-Parkinsonism. Both *DYT12* and *AHC* mutations lead to reduced ATPase activity, whereas *AHC* mutations did not affect the protein expression level that was observed for *DYT12*. Recently, a third allelic disorder for *ATP1A3* was identified, episodic Cerebellar Ataxia, Areflexia, Optic Atrophy, and Sensorineural Hearing Loss (CAOS) by exome sequencing (45). How different mutations in *ATP1A3* can lead to three disorders with distinct neurological manifestations is not yet known and needs further functional investigations but highlights the complexity and challenges of current genetics research.

### X-Linked Dystonia-Parkinsonism (Lubag)

X-linked dystonia-Parkinsonism (DYT3) is a recessive condition characterized by focal dystonia that is later followed by Parkinsonism. This rare condition is mainly prevalent in the Philippines and affects only males. Several disease-specific single-nucleotide changes (DSCs) and a small deletion were detected within TAF1 RNA polymerase II, TATA box-binding protein-associated factor, 250 kDa (*TAF1*) (46). Makino et al. showed that some of these variants are associated with reduced neuron-specific TAFI expression (47) that may underlie altered expression of genes involved in vesicular transport and dopamine metabolism fitting well with the known molecular pathways involved in the etiology of dystonia. The question remains if *TAF1* is indeed the DYT3 disease gene and transgenic mouse models carrying the various genetic variations in *Taf1* are needed to answer this question.

## Discussion and Future Perspectives

While the abovementioned challenges of validating pathogenic variants and establishing robust genotype–phenotype correlations do not refute the validity of the original findings, it should stress the importance of replication of genetic findings. Particularly in the setting of genetic counseling of patients and their families, all genetic findings have to be treated with caution until unequivocal proof of pathogenicity is available. It is likely that over the coming years, dozens, maybe hundreds of genes, will be nominated as potential disease or risk genes for dystonia. Their functional validation will be a major challenge for neurology, genetics, and clinical neuroscience.

Despite these caveats, the identification of more genes causing different forms of dystonia will allow to construct an increasingly complex network of cellular pathways that promises not only to eventually provide a better understanding of the cause(s) of dystonia, hopefully leading to new and better treatments, but may help us to understand the functions of sensory motor integration of the human brain on a molecular level.

## Author Contributions

DV and TG contributed equally to the writing of this review.

## Conflict of Interest Statement

The authors declare that the research was conducted in the absence of any commercial or financial relationships that could be construed as a potential conflict of interest.

## Footnotes

^1^http://evs.gs.washington.edu/EVS/.

^2^http://exac.broadinstitute.org/.

^3^http://genetics.bwh.harvard.edu/pph2/.

^4^http://sift.jcvi.org/.

^5^http://www.mutationtaster.org/.

## References

[1] Ozelius LJ Hewett JW Page CE Bressman SB Kramer PL Shalish C The early-onset torsion dystonia gene (DYT1) encodes an ATP-binding protein. Nat Genet (1997) 17:40–8. 10.1038/ng0997-40 9288096

[2] Zimprich A Grabowski M Asmus F Naumann M Berg D Bertram M Mutations in the gene encoding epsilon-sarcoglycan cause myoclonus-dystonia syndrome. Nat Genet (2001) 29:66–9. 10.1038/ng709 11528394

[3] Balint B Bhatia KP. Dystonia: an update on phenomenology, classification, pathogenesis and treatment. Curr Opin Neurol (2014) 27:468–76. 10.1097/WCO.0000000000000114 24978640

[4] Albanese A Sorbo FD Comella C Jinnah HA Mink JW Post B Dystonia rating scales: critique and recommendations. Mov Disord (2013) 28:874–83. 10.1002/mds.25579 23893443 PMC4207366

[5] Lupski JR Belmont JW Boerwinkle E Gibbs RA. Clan genomics and the complex architecture of human disease. Cell (2011) 147:32–43. 10.1016/j.cell.2011.09.008 21962505 PMC3656718

[6] MacArthur DG Manolio TA Dimmock DP Rehm HL Shendure J Abecasis GR Guidelines for investigating causality of sequence variants in human disease. Nature (2014) 508:469–76. 10.1038/nature13127 24759409 PMC4180223

[7] Ran FA Hsu PD Wright J Agarwala V Scott DA Zhang F. Genome engineering using the CRISPR-Cas9 system. Nat Protoc (2013) 8:2281–308. 10.1038/nprot.2013.143 24157548 PMC3969860

[8] Klein C Genetics in dystonia. Parkinsonism Relat Disord (2014) 20(Suppl 1):S137–42. 10.1016/S1353-8020(13)70033-6 24262166

[9] Fuchs T Saunders-Pullman R Masuho I Luciano MS Raymond D Factor S Mutations in GNAL cause primary torsion dystonia. Nat Genet (2013) 45:88–92. 10.1038/ng.2496 23222958 PMC3530620

[10] Charlesworth G Plagnol V Holmström KM Bras J Sheerin U-M Preza E Mutations in ANO3 cause dominant craniocervical dystonia: ion channel implicated in pathogenesis. Am J Hum Genet (2012) 91:1041–50. 10.1016/j.ajhg.2012.10.024 23200863 PMC3516598

[11] Saunders-Pullman R Fuchs T San Luciano M Raymond D Brashear A Ortega R Heterogeneity in primary dystonia: lessons from THAP1, GNAL, and TOR1A in Amish-Mennonites. Mov Disord (2014) 29:812–8. 10.1002/mds.25818 24500857 PMC4013240

[12] Ma L-Y Wang L Yang Y-M Feng T Wan X-H Mutations in ANO3 and GNAL gene in thirty-three isolated dystonia families. Mov Disord (2015) 30:743–4. 10.1002/mds.26190 25847575

[13] Xiao J Uitti RJ Zhao Y Vemula SR Perlmutter JS Wszolek ZK Mutations in CIZ1 cause adult onset primary cervical dystonia. Ann Neurol (2012) 71:458–69. 10.1002/ana.23547 22447717 PMC3334472

[14] Zech M Lam DD Francescatto L Schormair B Salminen AV Jochim A Recessive mutations in the α3 (VI) collagen gene COL6A3 cause early-onset isolated dystonia. Am J Hum Genet (2015) 96:883–93. 10.1016/j.ajhg.2015.04.010 26004199 PMC4457951

[15] Dufke C Hauser A-K Sturm M Fluhr S Wächter T Leube B Mutations in CIZ1 are not a major cause for dystonia in Germany. Mov Disord (2015) 30:740–3. 10.1002/mds.26198 25778706

[16] Nalls MA Pankratz N Lill CM Do CB Hernandez DG Saad M Large-scale meta-analysis of genome-wide association data identifies six new risk loci for Parkinson’s disease. Nat Genet (2014) 46:989–93. 10.1038/ng.3043 25064009 PMC4146673

[17] Lambert J-C Heath S Even G Campion D Sleegers K Hiltunen M Genome-wide association study identifies variants at CLU and CR1 associated with Alzheimer’s disease. Nat Genet (2009) 41:1094–9. 10.1038/ng.439 19734903

[18] International Multiple Sclerosis Genetics Consortium Lill CM Schjeide B-MM Graetz C Ban M Alcina A MANBA, CXCR5, SOX8, RPS6KB1 and ZBTB46 are genetic risk loci for multiple sclerosis. Brain (2013) 136:1778–82. 10.1093/brain/awt101 23739915 PMC3673463

[19] Lohmann K Schmidt A Schillert A Winkler S Albanese A Baas F Genome-wide association study in musician’s dystonia: a risk variant at the arylsulfatase G locus? Mov Disord (2014) 29:921–7. 10.1002/mds.25791 24375517

[20] Sardiello M Annunziata I Roma G Ballabio A. Sulfatases and sulfatase modifying factors: an exclusive and promiscuous relationship. Hum Mol Genet (2005) 14:3203–17. 10.1093/hmg/ddi351 16174644

[21] Abitbol M Thibaud J-L Olby NJ Hitte C Puech J-P Maurer M A canine Arylsulfatase G (ARSG) mutation leading to a sulfatase deficiency is associated with neuronal ceroid lipofuscinosis. Proc Natl Acad Sci U S A (2010) 107:14775–80. 10.1073/pnas.0914206107 20679209 PMC2930459

[22] Kowalewski B Lamanna WC Lawrence R Damme M Stroobants S Padva M Arylsulfatase G inactivation causes loss of heparan sulfate 3-O-sulfatase activity and mucopolysaccharidosis in mice. Proc Natl Acad Sci U S A (2012) 109:10310–5. 10.1073/pnas.1202071109 22689975 PMC3387061

[23] Nibbeling E Schaake S Tijssen MA Weissbach A Groen JL Altenmüller E Accumulation of rare variants in the arylsulfatase G (ARSG) gene in task-specific dystonia. J Neurol (2015) 262:1340–3. 10.1007/s00415-015-7718-3 25825126

[24] Mok KY Schneider SA Trabzuni D Stamelou M Edwards M Kasperaviciute D Genomewide association study in cervical dystonia demonstrates possible association with sodium leak channel. Mov Disord (2014) 29:245–51. 10.1002/mds.25732 24227479 PMC4208301

[25] Gómez-Garre P Huertas-Fernández I Cáceres-Redondo MT Alonso-Canovas A Bernal-Bernal I Blanco-Ollero A Lack of validation of variants associated with cervical dystonia risk: a GWAS replication study. Mov Disord (2014) 29:1825–8. 10.1002/mds.26044 25256078

[26] Bressman SB Sabatti C Raymond D de Leon D Klein C Kramer PL The DYT1 phenotype and guidelines for diagnostic testing. Neurology (2000) 54:1746–52. 10.1212/WNL.54.9.1746 10802779

[27] Gambarin M Valente EM Liberini P Barrano G Bonizzato A Padovani A Atypical phenotypes and clinical variability in a large Italian family with DYT1-primary torsion dystonia. Mov Disord (2006) 21:1782–4. 10.1002/mds.21056 16874761

[28] Risch NJ Bressman SB Senthil G Ozelius LJ. Intragenic Cis and Trans modification of genetic susceptibility in DYT1 torsion dystonia. Am J Hum Genet (2007) 80:1188–93. 10.1086/518427 17503336 PMC1867106

[29] Dobričić V Kresojević N Žarković M Tomić A Marjanović A Westenberger A Phenotype of non-c.907_909delGAG mutations in TOR1A: DYT1 dystonia revisited. Parkinsonism Relat Disord (2015) 21:1256–9. 10.1016/j.parkreldis.2015.08.001 26297380

[30] Asmus F Gasser T Inherited myoclonus-dystonia. Adv Neurol (2004) 94:113–9. 14509663

[31] Müller B Hedrich K Kock N Dragasevic N Svetel M Garrels J Evidence that paternal expression of the epsilon-sarcoglycan gene accounts for reduced penetrance in myoclonus-dystonia. Am J Hum Genet (2002) 71:1303–11. 10.1086/344531 12444570 PMC378568

[32] Asmus F Hjermind LE Dupont E Wagenstaller J Haberlandt E Munz M Genomic deletion size at the epsilon-sarcoglycan locus determines the clinical phenotype. Brain (2007) 130:2736–45. 10.1093/brain/awm209 17898012

[33] Kuivaniemi H Tromp G Prockop DJ. Mutations in collagen genes: causes of rare and some common diseases in humans. FASEB J (1991) 5:2052–60. 2010058 10.1096/fasebj.5.7.2010058

[34] Grimes DA Han F Lang AE St George-Hyssop P Racacho L Bulman DE. A novel locus for inherited myoclonus-dystonia on 18p11. Neurology (2002) 59:1183–6. 10.1212/WNL.59.8.1183 12391345

[35] Groen JL Andrade A Ritz K Jalalzadeh H Haagmans M Bradley TEJ CACNA1B mutation is linked to unique myoclonus-dystonia syndrome. Hum Mol Genet (2015) 24:987–93. 10.1093/hmg/ddu513 25296916 PMC4817404

[36] Mencacci NE Rubio-Agusti I Zdebik A Asmus F Ludtmann MHR Ryten M A missense mutation in KCTD17 causes autosomal dominant myoclonus-dystonia. Am J Hum Genet (2015) 96:938–47. 10.1016/j.ajhg.2015.04.008 25983243 PMC4457957

[37] Beuckmann CT Sinton CM Miyamoto N Ino M Yanagisawa M. N-type calcium channel alpha1B subunit (Cav2.2) knock-out mice display hyperactivity and vigilance state differences. J Neurosci (2003) 23:6793–7. 12890773 10.1523/JNEUROSCI.23-17-06793.2003 PMC6740709

[38] Jen J Yue Q Nelson SF Yu H Litt M Nutt J A novel nonsense mutation in CACNA1A causes episodic ataxia and hemiplegia. Neurology (1999) 53:34–7. 10.1212/WNL.53.1.34 10408533

[39] Mencacci NE R’bibo L Bandres-Ciga S Carecchio M Zorzi G Nardocci N The CACNA1B R1389H variant is not associated with myoclonus-dystonia in a large European multicentric cohort. Hum Mol Genet (2015) 24:5326–9. 10.1093/hmg/ddv255 26157024 PMC4550822

[40] Mencacci NE Isaias IU Reich MM Ganos C Plagnol V Polke JM Parkinson’s disease in GTP cyclohydrolase 1 mutation carriers. Brain (2014) 137:2480–92. 10.1093/brain/awu179 24993959 PMC4132650

[41] Wijemanne S Jankovic J Dopa-responsive dystonia – clinical and genetic heterogeneity. Nat Rev Neurol (2015) 11:414–24. 10.1038/nrneurol.2015.86 26100751

[42] Svetel M Djarmati A Dragasević N Savić D Culjković B Romac S SCA2 and SCA3 mutations in young-onset dopa-responsive parkinsonism. Eur J Neurol (2003) 10:597. 10.1046/j.1468-1331.2003.00671.x 12940846

[43] Paisán-Ruiz C Guevara R Federoff M Hanagasi H Sina F Elahi E Early-onset l-dopa-responsive parkinsonism with pyramidal signs due to ATP13A2, PLA2G6, FBXO7 and spatacsin mutations. Mov Disord (2010) 25:1791–800. 10.1002/mds.23221 20669327 PMC6005705

[44] de Carvalho Aguiar P Sweadner KJ Penniston JT Zaremba J Liu L Caton M Mutations in the Na+/K+-ATPase alpha3 gene ATP1A3 are associated with rapid-onset dystonia parkinsonism. Neuron (2004) 43:169–75. 10.1016/j.neuron.2004.06.028 15260953

[45] Heimer G Sadaka Y Israelian L Feiglin A Ruggieri A Marshall CR CAOS-episodic cerebellar ataxia, areflexia, optic atrophy, and sensorineural hearing loss: a third allelic disorder of the ATP1A3 gene. J Child Neurol (2015) 30:1749–56. 10.1177/0883073815579708 25895915

[46] Nolte D Niemann S Müller U Specific sequence changes in multiple transcript system DYT3 are associated with X-linked dystonia parkinsonism. Proc Natl Acad Sci U S A (2003) 100:10347–52. 10.1073/pnas.1831949100 12928496 PMC193564

[47] Makino S Kaji R Ando S Tomizawa M Yasuno K Goto S Reduced neuron-specific expression of the TAF1 gene is associated with X-linked dystonia-parkinsonism. Am J Hum Genet (2007) 80:393–406. 10.1086/512129 17273961 PMC1821114

